# Effectiveness and safety of vericiguat in patients with heart failure and reduced ejection fraction: a narrative review of real-world evidence studies

**DOI:** 10.3389/fcvm.2025.1644646

**Published:** 2025-11-06

**Authors:** Carlos Escobar, Pedro Caravaca-Pérez, Jose Maria Fernandez Rodriguez, Ines Gomez-Otero, Ainara Lozano-Bahamonde, Alejandro I. Pérez-Cabeza, José Pérez-Silvestre, Carles Rafols, Alberto Esteban-Fernández

**Affiliations:** 1 Cardiology Department, University Hospital La Paz, Madrid, Spain; 2 Heart Failure and Transplant Unit, Hospital Universitario Clinic, Barcelona, Spain; 3 Internal Medicine, Hospital Universitario Central de Asturias, Oviedo, Spain; 4 Heart Failure Unit, Department of Cardiology, Hospital Clínico, Universitario Santiago de Compostela, Santiago de Compostela, Spain; 5 Heart Failure Unit, Department of Cardiology, Hospital Universitario Basurto, Bilbao, Spain; 6 Department of Cardiology, Virgen de la Victoria University Hospital, CIBERCV, Malaga, Spain; 7 Internal Medicine Department, Consorci Hospital General Universitari de Valencia, Valencia, Spain; 8 Medical Department, Bayer Hispania, Barcelona, Spain; 9 Cardiology Department, Hospital Universitario Severo Ochoa, Leganés, Madrid, Spain

**Keywords:** heart failure, reduced ejection fraction, vericiguat, real-world evidence, VICTORIA trial

## Abstract

Heart failure is associated with a high risk of morbidity, mortality, and healthcare resource use. Its pathophysiology and treatment are complex and involve various neurohormonal systems. Early guideline-directed medical therapy is currently recommended in patients with heart failure with reduced ejection fraction; however, patients continue to be at high risk of rehospitalization and death. The VICTORIA clinical trial compared vericiguat, an oral soluble guanylate cyclase stimulator that restores the nitric oxide pathway, with placebo, added to guideline-recommended treatment. VICTORIA demonstrated a significant benefit compared to standard therapy. Nonetheless, it is essential to understand how vericiguat performs in real-world settings. A narrative literature review was performed to compare the findings of the VICTORIA trial and real-world evidence studies. Real-world evidence was analyzed from almost 6,000 patients. Vericiguat was shown to be associated with improvements in quality of life and New York Heart Association functional class, left ventricular reverse remodeling, and numerically lower rates of heart failure hospitalizations and mortality than in the VICTORIA trial, although it is used in older patients with more comorbidities. Moreover, real-world evidence studies showed these effects to be infrequent and similar to those reported in the VICTORIA trial, with low discontinuation rates, indicating that vericiguat was very well tolerated in a real-world population. We present the most comprehensive review to date on vericiguat in clinical practice, providing an overview of its effects on clinical, biochemical, and imaging parameters. Our findings suggest that vericiguat could be a component in the comprehensive management of heart failure with reduced ejection fraction. However, further specific investigations with longer follow-up and larger samples would enable us to resolve some of the hypotheses put forward in our study.

## Introduction

Heart failure (HF) is associated with a high risk of morbidity, mortality, and healthcare resource use (1). The current prevalence of HF is around 1%–3% with disparities between countries. However, it is estimated that this percentage will increase in the coming years (2, 3). The pathophysiology of HF is complex, involving various neurohormonal systems that should be targeted to reduce disease burden and progression (4, 5). In this context, early use of guideline-directed medical therapy (GDMT) is recommended in patients with HF with reduced ejection fraction (HFrEF). GDMT includes renin-angiotensin-aldosterone system (RAAS) inhibitors, especially angiotensin receptor-neprilysin inhibitors (ARNI), as well as beta blockers, mineralocorticoid receptor antagonists (MRA), and sodium-glucose cotransporter 2 inhibitors (SGLT2i) (6, 7).

However, despite these therapies, patients continue to be at high risk of rehospitalization or death due to HF (8, 9), mainly because HF comprises the pathophysiological mechanisms on which these drugs act, but also because of other mechanisms, such as oxidative stress, endothelial dysfunction, inflammation, and impairment of the guanylate cyclase system. Consequently, the response to therapy is only partial, and, although ARNI increase cyclic guanosine monophosphate (cGMP) levels, the guanylate cyclase system may not be completely restored (4, 5). By contrast, vericiguat is an oral soluble guanylate cyclase stimulator that restores the nitric oxide pathway in patients with HFrEF (5).

The VICTORIA trial demonstrated the risk of hospitalization due to HF and cardiovascular death in patients with symptomatic HFrEF and recently decompensated HF to be significantly lower with vericiguat than with placebo (10). Nonetheless, the information provided by clinical trials cannot necessarily be extended to the whole population with HF (11, 12). In fact, real-world evidence (RWE) studies have shown that only 20%–60% of patients with HFrEF meet the eligibility criteria of the VICTORIA trial (13, 14). As a result, understanding how vericiguat performs in real-world settings, where patient profiles and background therapy differ from those of clinical trials, is key to refining its positioning in the treatment of patients with HFrEF.

This study aimed to critically review RWE on use of vericiguat and to perform an indirect comparison with the findings of the VICTORIA trial and RWE studies.

## Methods

A narrative literature review based on a comprehensive search strategy for peer-reviewed articles was performed to identify literature published up to February 2025 in the PubMed and Embase databases (15, 16). A combination of controlled vocabulary and keywords was used to perform the search and included Heart failure, Worsening, Treatment, and Vulnerable period. The operators “AND” and “OR” were used to combine these concepts. The search included manuscripts in English and in Spanish, from journal articles and congress materials (2024 Spanish Society of Cardiology, 2024 European Society of Cardiology, and 2024 European HF Association of the European Society of Cardiology). All manuscripts providing data from RWE with vericiguat (excluding those limited to isolated clinical case reports) were analyzed, with no further limits. All citations were introduced into a citation management system, and duplicates were eliminated. Original data from the VICTORIA trial and observational studies were critically discussed. Indirect comparisons between RWE studies and the VICTORIA trial were performed.

The VICTORIA trial was a phase 3, randomized, double-blind clinical trial comprising 5,050 patients with chronic symptomatic HF (New York Heart Association class [NYHA] functional class II to IV), left ventricular ejection fraction (LVEF) <45%, elevated natriuretic peptides levels within 30 days prior to randomization, a previous hospitalization for HF within the 6 months prior to randomization, or intravenous diuretic treatment for HF (without hospitalization) within the 3 months prior to randomization. Patients were assigned to receive vericiguat (target dose, 10 mg once daily) or placebo, in addition to standard HF therapy. Data from the quality of life and echocardiographic substudies were also analyzed (10, 17, 18).

**Figure 1:**
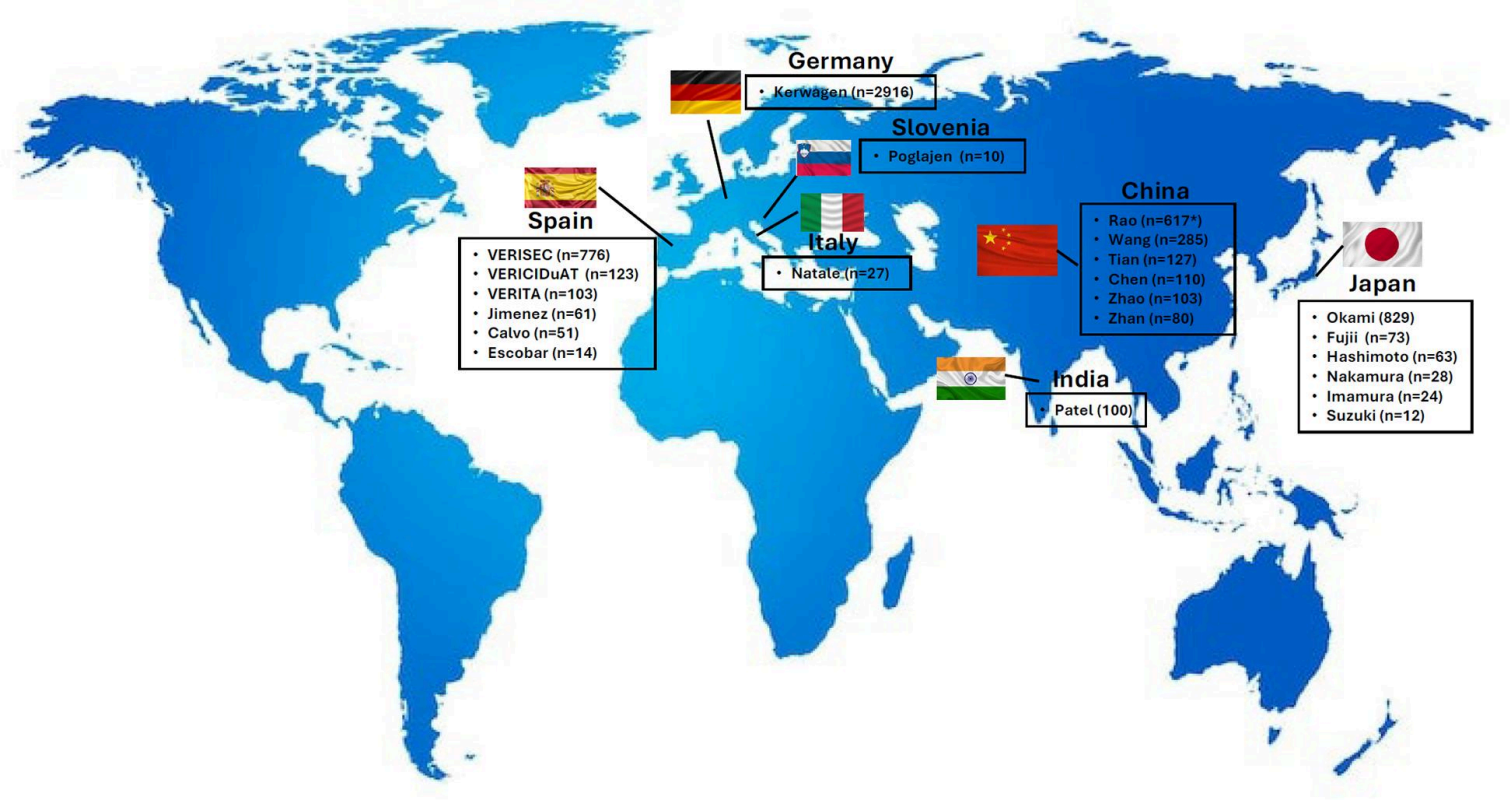
RWE studies with vericiguat by country. RWE, real-world evidence. Figure based on data from references (19–31, 32, 33–40).

A total of 22 RWE studies were included in this review, as follows: 6 from Spain (19–24), 6 from Japan (25–30), 6 from China (31–36), 1 from Germany (37), 1 from Italy (38), 1 from Slovenia (39), and 1 from India (40) (Figure 1). The design of the studies, as well as the outcome measures analyzed are summarized in Table 1.

**Table 1:** Design and study outcomes of the RWE studies with vericiguat.

Study (year of publication)	Country	Design	Study outcomes (variables and events)
Calvo (2024) (19)	Spain	Retrospective cohort study that included patients from a single center who had started vericiguat between September 2021 and February 2024.	Baseline clinical characteristics. NYHA functional class. LVEF. Natriuretic peptides. Decompensated HF and mortality. Safety.
Jimenez (2024)	Spain	Prospective cohort study of patients who started treatment with vericiguat in 8 hospitals. Laboratory and clinical variables were compared at baseline and after 6 months of follow-up.	Baseline clinical characteristics. NYHA functional class. Decompensated HF and mortality. Safety
VERISEC (2025)	Spain	Multicenter, prospective, cross-sectional registry which included patients from 43 centers between December 2022 and October 2023. Patients were followed up for 12 months after starting treatment with vericiguat (85.8% started treatment in the outpatient clinic after decompensation and 14.2% during hospitalization).	Baseline clinical characteristics vs. VICTORIA trial
VERICIDuAT (2025)	Spain	Observational, retrospective, longitudinal study of patients treated with vericiguat in a tertiary hospital according to clinical criteria and followed for an average of 162 days.	Baseline clinical characteristics. NYHA functional class. Decompensated HF and mortality. Natriuretic peptides. Safety.
VERITA (2024)	Spain	Prospective and observational cohort study of patients with HFrEF and recent HF worsening episode requiring intravenous therapy who initiated vericiguat in an HF outpatient clinic.	Baseline clinical characteristics. NYHA functional class, EQ-5D, VAS. Decompensated HF and mortality. Safety.
		A subanalysis was performed In patients with > 6-month follow-up to assess the impact of vericiguat on functional class, quality of life, readmissions, and mortality (median follow-up of 303 days).	
Escobar (2025) (24)	Spain	Retrospective analysis of patients with HFrEF and an implantable cardioverter-defibrillator who started treatment with vericiguat in January 2023 at a tertiary university hospital.	Baseline clinical characteristics. LVEF. Natriuretic peptides. Ventricular arrhythmias. Decompensated HF and mortality. Safety.
Okami (2024) (25)	Japan	Retrospective cohort study using a nationwide hospital administrative database, Medical Data Vision, which included patients with HF who were prescribed vericiguat between July 1, 2021, and September 30, 2022. Patients were followed up for 90 days after starting vericiguat.	Baseline clinical characteristics. Mean pulmonary artery pressure and pulmonary artery interlocking pressure.
Imamura (2024) (26)	Japan	Retrospective study that involved patients undergoing 3-month vericiguat therapy alongside complete quadruple medical therapy to assess factors associated with changes in serum NT pro-B-type natriuretic peptide levels during the follow-up.	Baseline clinical characteristics.
Suzuki (2024) (27)	Japan	Prospective study that included patients who experienced a worsening of HFrEF in hospital and were followed for 3 months after starting vericiguat (March-December 2022).	Baseline clinical characteristics.
Fujii (2024) (28)	Japan	Prospective study of 5 hospitals that evaluated the effects of vericiguat on LV reverse remodeling in patients who started treatment with vericiguat between October 2021 and September 2023, with a follow-up of 6 months.	Baseline clinical characteristics. LVEF. Decompensated HF and mortality.
Hashimoto (2024) (29)	Japan	Retrospective analysis of consecutive patients who received vericiguat for the treatment of HFrEF at a single hospital between 2021 and 2023. The mean follow-up was 266 days, and vericiguat was initiated during hospitalization in 34.9% and in the outpatient clinic in 65.1% of patients.	Baseline clinical characteristics. LVEF. Natriuretic peptides.
Nakamura (2023) (30)	Japan	Retrospective analysis of consecutive patients who received vericiguat between September 2021 and December 2022 and were followed for a median of 236 days.	Baseline clinical characteristics. Natriuretic peptides. Safety.
Chen (2024) (31)	China	Prospective single-center study of 110 patients admitted with acute HF and followed for 180 days. The incidence of HF-associated events was analyzed according to the use of guideline-directed medical therapy.	Decompensated HF and mortality.
Tian (2024) (32)	China	Multicenter and observational prospective cohort study that included 200 HFrEF patients in China (127 treated with vericiguat) and followed up for 6 months.	Baseline clinical characteristics. LVEF. Natriuretic peptides. Safety.
Zhao (2024) (33)	China	Patients with HF treated with vericiguat in hospital were consecutively included from December 1, 2022 to February 1, 2024.	Baseline clinical characteristics. MLHFQ score. LVEF. Natriuretic peptides.
Rao (2024) (34)	China	The adverse event reports associated with vericiguat from 2021Q1 to 2024Q1 were analyzed using data from the Food and Drug Administration Adverse Event Reporting System.	Safety.
Wang (2024) (35)	China	Multicenter study that included patients with HF who initiated treatment with vericiguat from September 2022 to August 2023 in 9 hospitals. Patients were evaluated at baseline and after 1 year of treatment. Vericiguat was initiated during hospitalization in 78.2% of patients. Only 44.9% met the VICTORIA trial inclusion criteria.	Baseline clinical characteristics.
Zhan (2025) (36)	China	Prospective observational study that consecutively included patients with HFrEF admitted to hospital who started treatment with vericiguat between May 2022 and May 2023 and were followed for 6 months.	Baseline clinical characteristics. Weber Functional Classification. LVEF. Peak oxygen consumption, anaerobic threshold, carbon dioxide equivalent slope. Decompensated HF and mortality. Safety.
Kerwagen (2024) (37)	Germany	Retrospective analysis of longitudinally collected data from the IQVIA™ LRx database, including patients who initiated vericiguat between September 2021 and December 2022.	Baseline clinical characteristics.
Natale (2024) (38)	Italy	Retrospective study that analyzed the effects of vericiguat on renal arterial resistance index in 27 HF patients.	Baseline clinical characteristics. Renal arterial resistance index.
Poglajen (2024) (39)	Slovenia	Prospective nonrandomized study that included 10 patients with chronic HF and LV assist device support treated with vericiguat.	Right ventricular function.
Patel (2024) (40)	India	Observational cohort study that involved 100 patients with symptomatic HFrEF recruited from a tertiary care hospital. Patients were divided into 2 groups according to the use of vericiguat. The risk of mortality, HF admissions, and changes in ejection fraction over 6 months were evaluated.	Baseline clinical characteristics. NYHA functional class. LVEF. Decompensated HF and mortality. Safety.

EQ-5D, EuroQol-5D; HF, heart failure; HFrEF, heart failure with reduced ejection fraction; LVEF, left ventricular ejection fraction; MLHFQ, Minnesota Living with Heart Failure Questionnaire; NYHA New York Heart Association; VAS, visual analog scale.

Table based on data from references (19–40).

Nine studies had a retrospective design (19, 22, 24–26, 29, 30, 37, 38), and 12 studies had a prospective design (20, 21, 23, 27, 28, 31–33, 35, 36, 39, 40). Rao et al. (34) analyzed vericiguat-related adverse events using data from the Food and Drug Administration Adverse Event Reporting System. Overall, 14 studies included patients from a single center (19, 22–24, 26, 27, 29–31, 33, 36, 38–40), and the remaining 8 were multicenter studies (20, 21, 25, 28, 32, 34, 35, 37).

Most of the RWE studies provided baseline clinical characteristics, including HF treatments, as well as the VICTORIA baseline characteristics (vericiguat arm) (10, 19–30, 32, 33, 35–38, 40). Changes in quality of life and functional class after treatment with vericiguat were analyzed in the VICTORIA trial and in 7 RWE studies (10, 17, 19, 20, 22, 23, 33, 36, 40). The evolution of hospitalizations for HF and mortality after treatment with vericiguat were analyzed in the VICTORIA trial and in 9 RWE studies (10, 19, 20, 22–24, 28, 31, 36, 40). Changes in heart structure and function were assessed in the VICTORIA trial and in 9 RWE studies during treatment with vericiguat (18, 19, 24, 28, 29, 32, 33, 36, 39, 40). The evolution of natriuretic peptides (BNP or NT-proBNP) with vericiguat was reported in 7 RWE studies (19, 22, 24, 29, 30, 32, 33). Furthermore, changes in HF treatments after the start of vericiguat were specifically analyzed in 3 RWE studies (23, 25, 37), as were potential benefits with vericiguat (19, 22, 24, 27, 36, 38).

Finally, the safety profile of vericiguat (the proportion of patients attaining the 10 mg dose and adverse effect and discontinuation rates) was also analyzed in the VICTORIA trial and in 12 RWE studies (10, 19, 20, 22–24, 28, 30, 32, 34, 36, 37, 40).

All data were reported on a descriptive basis, with the mean or the median in the case of quantitative variables and percentages in the case of qualitative variables. Only indirect comparisons were made.

## Results

Regarding the baseline clinical characteristics in the VICTORIA trial (2,526 patients treated with vericiguat), mean age was 67.3 years, 76.1% were men, mean systolic blood pressure was 121.2 mmHg, mean LVEF was 28.9%, and median NT-proBNP was 2,816 pg/ml. NYHA functional classes II and III were recorded in 59.0% and 39.7% of patients, respectively. Therapy was with RAAS inhibitors in 87.9% (14.5% ARNI), beta blockers in 93.1%, MRA in 70.3%, and SGLT2i in only 2.7%. In the RWE studies, the number of patients included varied widely, from 12 to 2,916. Age ranged from 51.0 to 78.2 years, the percentage of men from 53.7% to 84.1%, mean systolic blood pressure from 99.6 to 124.0 mmHg, LVEF from 19% to 35%, and NT-proBNP from 549.4 to 7,055 pg/ml. In all the studies, most patients had NYHA functional class II or III, although the distribution was heterogeneous. In general, the proportion of patients treated with GDMT was high (Table 2) (10, 19–30, 32, 33, 35–38, 40).

**Table 2:** Baseline clinical characteristics of the VICTORIA trial (VERICIGUAT arm) and RWE studies with vericiguat.

Study	VICTORIA (*n* = 2,526)	Kerwagen (*n* = 2,916)	Okami (*n* = 829)	VERISEC (*n* = 776)	Wang (*n* = 285)	Tian (*n* = 127)	VERICIDuAT (*n* = 123)	VERITA (*n* = 103)	Zhao (*n* = 103)	Patel (*n* = 100)	Zhan (*n* = 80)	Fujii (*n* = 73)	Hashimoto (*n* = 63)	Jimenez (*n* = 61)	Calvo (*n* = 51)	Nakamura (*n* = 28)	Natale (*n* = 27)	Imamura (*n* = 24)	Escobar (*n* = 14)	Suzuki (*n* = 12)
Biodemographic data																				
Age, years	67.5	73.0	75.5	72.4	64.8	51.0	78.2	71.3	56	67.3	56.9	71.9	54.3	69.7	71.7	66	<65 33.3%; 65–75 48.1% > 75 18.5%	66	77.0	63
Male sex %	76.0	66.3	69.0	79.6	77.2	72.7	61.8	72.8	78.6	62.5	53.7	76	74.6	84.1	74.5	82	59.3	83	71.4	83.3
SBP, mmHg	121.2			117.1	124.0	107.1	118.7	117	116.8			108.9	99.6	115.2		102	123			101.5
Comorbidities																				
Hypertension, %	79.3		91.7	75.6	34.0	27.6	74.8	86.4	33.0	72.9	32.5	53							78.6	
Diabetes, %	48.6		60.1	53.0	11.6	14.2	48.8	56.3	15.5	45.8	35.0	35				11		38	42.9	
AF, %	50.6		32.8	49.6	18.6	11.8		66.0			71.2	48	36.5			14		42	78.6	
CAD, %	59.8		71.3		36.8	24.4		56.3			41.2							13	64.3	
HF data																				
HF etiology, %																				
Ischemic				49.6				56.3				51	22.2	61.7	58.8	11		13		25
Dilated				29.2				33.0					42.9			36		38		75
LVEF, %	29.0			30.1	34.9	31.0	35			32.5	31.2	31.8	29.9	26.2	30.9	33	35	34	32.1	19
NYHA, %																				
I	<0.1			3.2	1		9.2					7								
II	58.6			57.6	4.2	60.9	53.0	40.8	44.7			46	44.4	34.9	58.8	54	37.0			50
III	40.0			37.5	55.1	32.0	32.8	59.2	50.5	58.3 (III/IV)		42	47.6	52.4	39.2	32	63.0			16.7
IV	1.4			1.4	39.6	6.3	5.0		4.8			5	7.9			14				33.3
BNP, pg/ml												427	525.3			190				419
NT-proBNP, pg/ml	2,803.5			3,551.0	2,915.0	695.4	7,055	2,034			549.4	4,773		4,666.7	3,247	903		877		
HF treatment																				
RAASi, %	87.6	74.6	79.5		72.2							62			96.1	96	100	100	92.9	100
ACEI/ARB	73.3	21.8	35.6	83.5	3.9	90.6						27	93.7			14	0	0	28.6	50
ARNI	14.3	52.8	43.9		68.3		70.7	96.1	89.3		76.2	35				82	100	100	64.3	50
Beta blockers, %	93.2	75.7	74.3	90.5	74.3	89.8	87.8	99.0	86.4		53.7	81	90.5		94.1	96		100	71.4	83.3
MRA, %	69.3	52.1	53.9	78.1	84.8	88.2	59.3	90.3	87.4		86.2	76	82.5		84.3	89		100	100	100
SGLT2i, %	2.7	50.7	54.4	91.8	68.7	89.0	87.8	96.1	90.3		31.2	70	79.4		80.3	79	100	100	71.4	100

ACEI, angiotensin-converting-enzyme inhibitors; AF, atrial fibrillation/flutter; ARB, angiotensin II receptor blockers; ARNI, angiotensin receptor-neprilysin inhibitor; BNP, brain natriuretic peptide; CAD, coronary artery disease; HF, heart failure; LVEF, left ventricular ejection fraction; MRA, mineralocorticoid receptor antagonists; NT-proBNP, N-terminal prohormone of brain natriuretic peptide; NYHA, New York Heart Association; SBP, systolic blood pressure; RAASi, renin angiotensin system inhibitors; RWE, real-world evidence; SGLT2i, sodium/glucose cotransporter 2 inhibitors.

Table based on data from references (10, 19–30, 32, 33, 35–38, 40).

**Figure 2:**
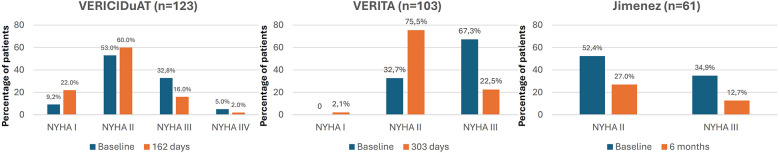
Changes in NYHA functional class after treatment with vericiguat. NYHA, New York heart association. Figure based on data from references (20, 22, 23).

With respect to quality of life and functional class, the VICTORIA trial showed that, while the relative benefits of vericiguat over placebo were independent of baseline health status, no significant differences were observed in the Kansas City Cardiomyopathy Questionnaire scores between the vericiguat and placebo groups during the study. In the RWE studies that specifically analyzed quality of life and functional class, an improvement was observed in NYHA functional class and in quality of life (Figure 2, Supplementary Table S1) (10, 17, 19, 20, 22, 23, 33, 36, 40).

**Figure 3:**
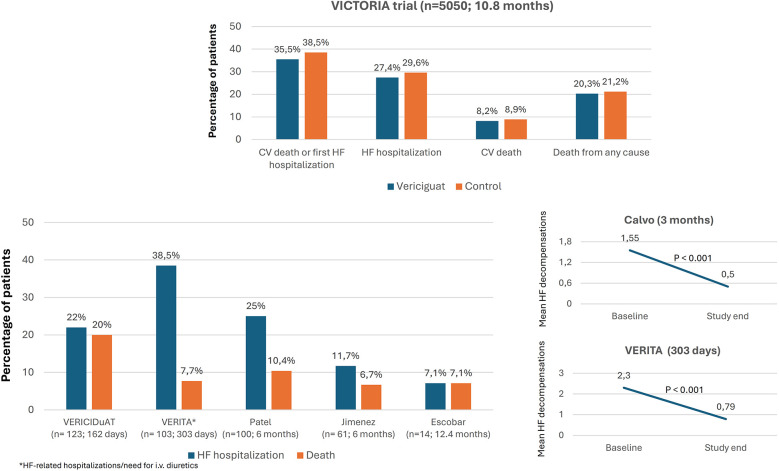
Evolution of hospitalizations for heart failure and mortality after treatment with vericiguat. CV, cardiovascular; HF, heart failure, (*n*: number of patients treated with vericiguat; follow-up). Figure based on data from references (10, 19, 20, 22–24, 40).

In the VICTORIA trial, over a median follow-up of 10.8 months, the primary outcome event (a composite of cardiovascular death or first HF hospitalization) was recorded in 35.5% of patients treated with vericiguat, death from cardiovascular causes in 16.4%, and hospitalization for decompensated HF in 27.4%. Nine RWE studies analyzed HF and mortality events during the follow-up. In general, rates of hospitalizations for HF and mortality tended to be lower than in the VICTORIA trial. However, the inclusion criteria and the follow-up periods differed between the studies (Figure 3, Supplementary Table S2) (10, 19, 20, 22–24, 28, 31, 36, 40).

**Figure 4:**
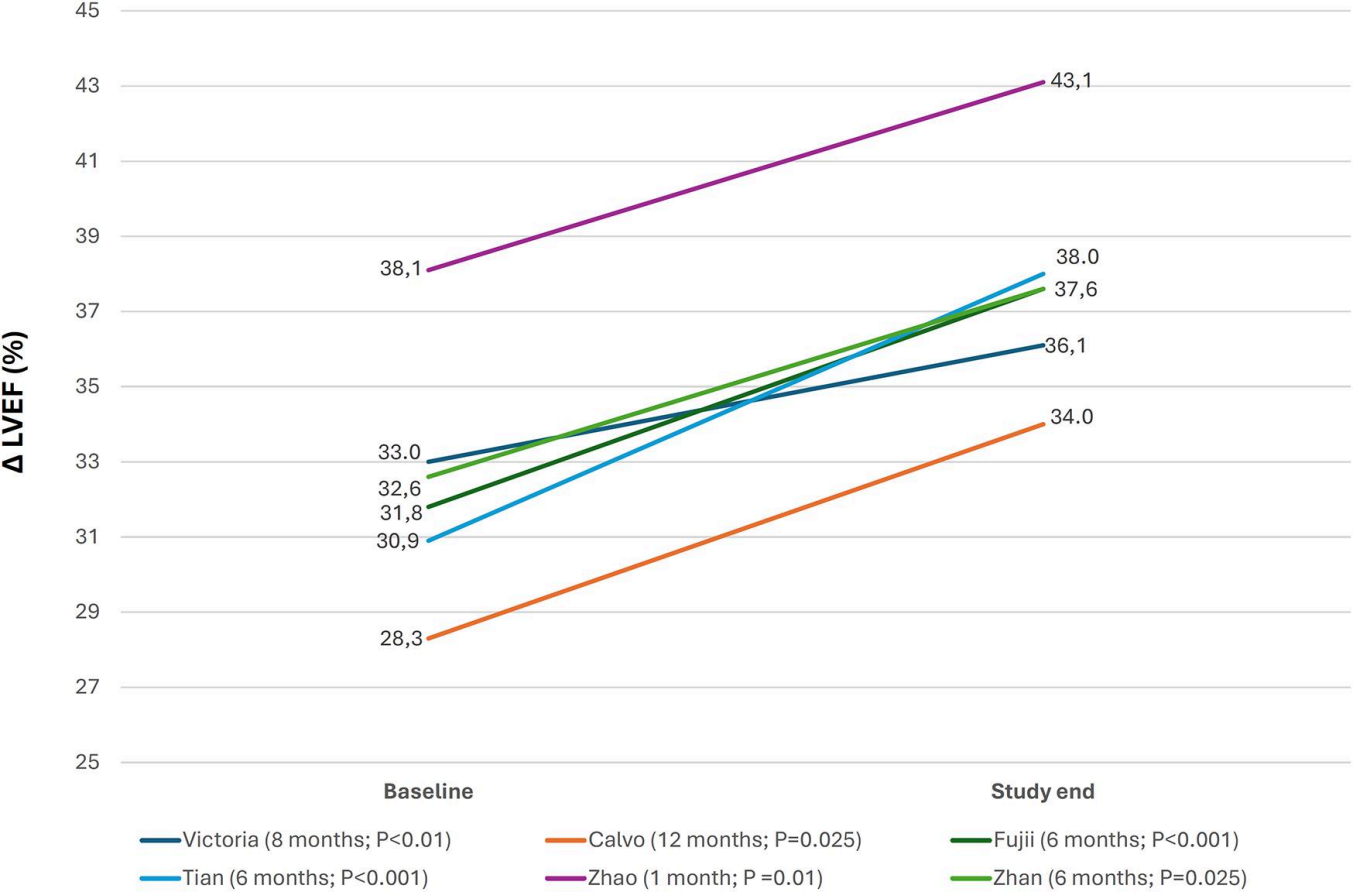
Changes in left ventricular ejection fraction after treatment with vericiguat. *Δ*LVEF, change in left ventricular ejection fraction from baseline to study end, (follow-up; *P* value study end-baseline). Figure based on data from references (18, 19, 28, 32, 33, 36).

**Figure 5:**
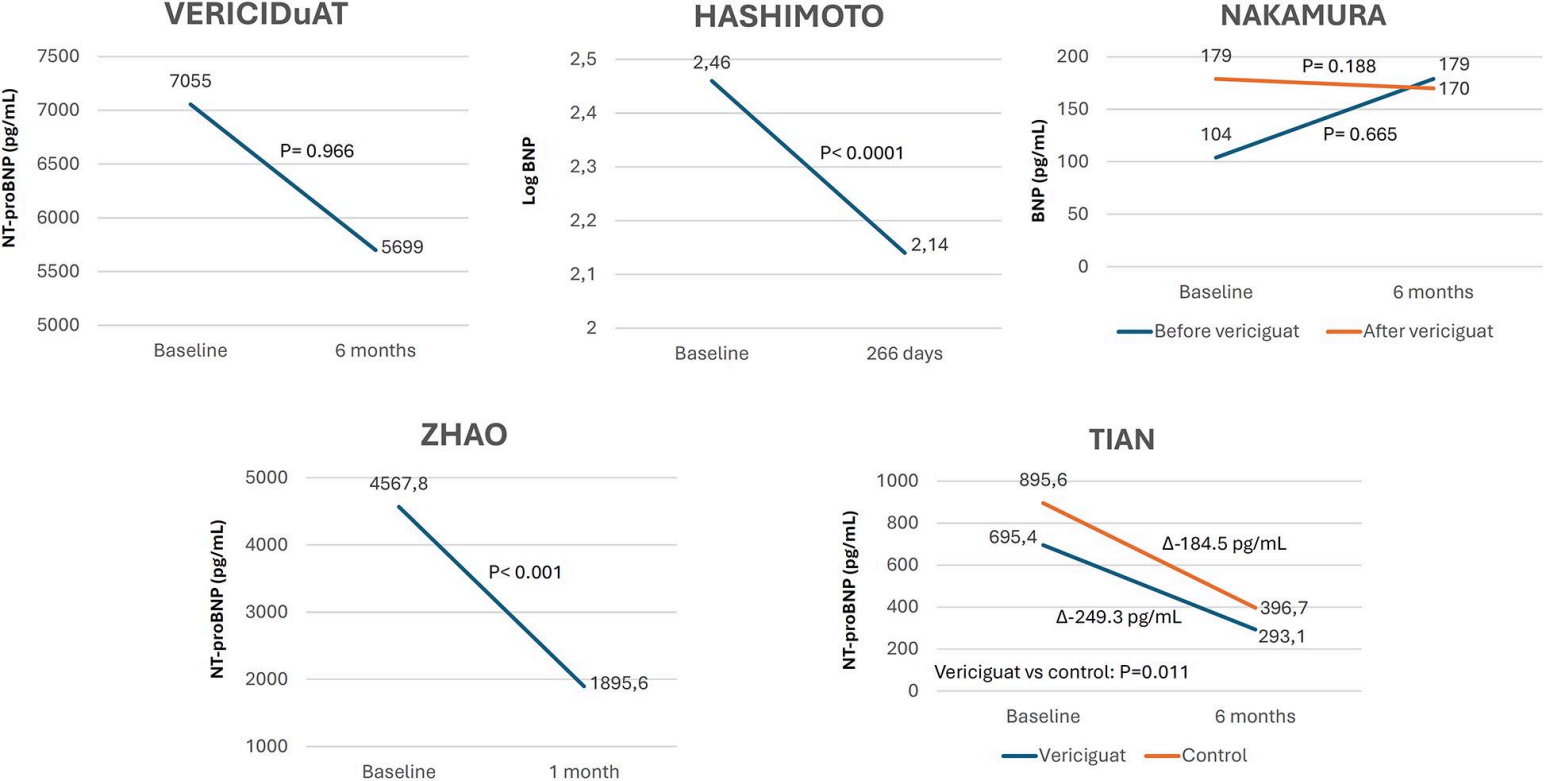
Changes in natriuretic peptides levels after treatment with vericiguat. Figure based on data from references (22, 29, 30, 32, 33).

The prespecified echocardiographic substudy of the VICTORIA trial showed a significant improvement in LV structure and function, suggesting LV reverse remodeling with vericiguat. This was confirmed by most RWE studies in which changes in echocardiographic parameters were analyzed (Figure 4, Supplementary Table S3) (18, 19, 24, 28, 29, 32, 33, 36, 39, 40). Around half of the studies showed a significant reduction in natriuretic peptide levels with vericiguat, whereas the remainder showed a neutral effect (Figure 5, Supplementary Table S3) (19, 22, 24, 29, 30, 32, 33). In addition, treatment with vericiguat enabled optimization of concomitant HF drugs (Supplementary Table S3) (23, 25, 37).

**Figure 6:**
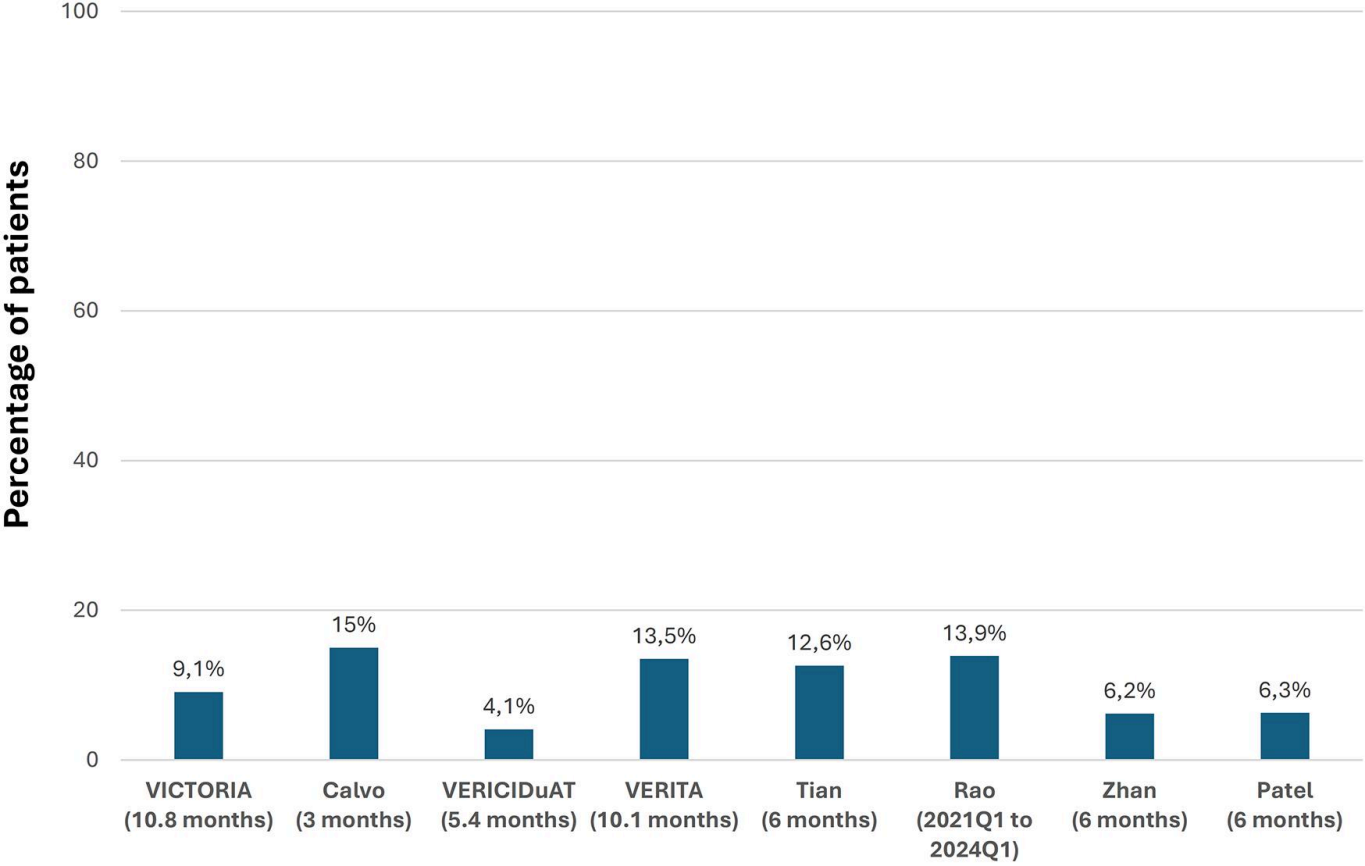
Percentage of patients with symptomatic hypotension during treatment with vericiguat. (Follow-up) Figure based on data from references (10, 19, 22, 23, 32, 34, 36, 40).

**Figure 7:**
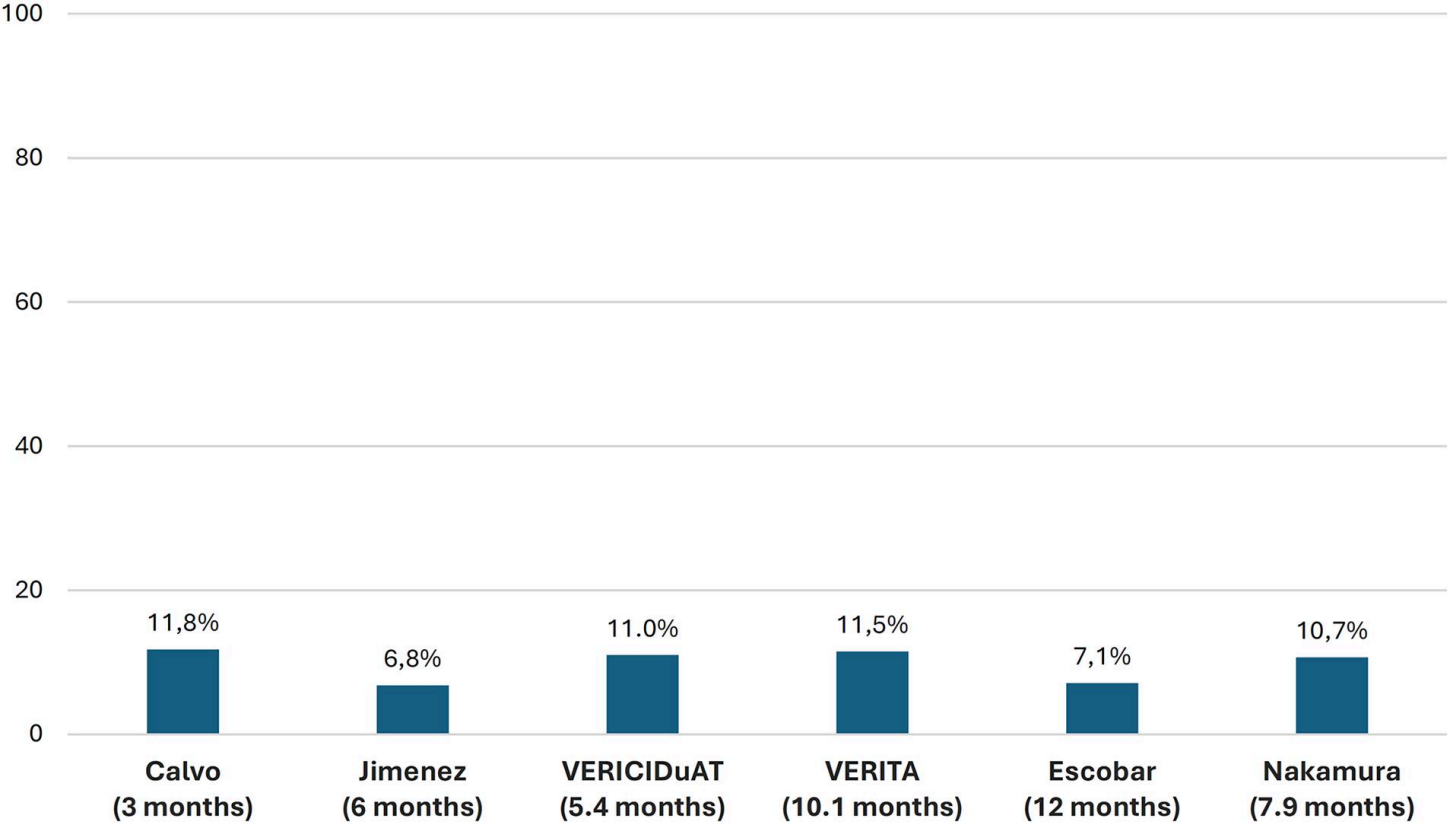
Discontinuation rates in RWE studies. RWE, real-world evidence, (follow-up). Figure based on data from references (19, 20, 22–24, 30).

In the VICTORIA trial, 90.3% of patients achieved the 10-mg target dose of vericiguat. However, in RWE studies, this proportion ranged from 50% to 80%. Regarding adverse effects, 32.8% of patients in the VICTORIA trial presented at least 1 adverse event, although only 1.6% experienced serious adverse events and 9.1% presented with symptomatic hypotension. In the RWE studies, adverse effects were infrequent, occurring at a similar rate to the VICTORIA trial, with symptomatic hypotension being the most relevant. The discontinuation rates of vericiguat in RWE studies ranged from 6.8% to 11.8% after 3–12 months of follow-up (Figures 6, 7 and Supplementary Table S4) (10, 19, 20, 22–24, 28, 30, 32, 34, 36, 37, 40).

## Discussion

This is the most comprehensive review to date on vericiguat in clinical practice, summarizing its impact on clinical, biochemical, and imaging outcomes. This analysis of RWE was performed in almost 6,000 patients and showed that vericiguat is being used in patients older than those included in the VICTORIA trial but with better background HF therapy, including devices. In real-world practice, vericiguat has been associated with improvements in quality of life and NYHA functional class, LV reverse remodeling, and numerically lower rates of hospitalizations for HF and mortality.

RWE is essential when attempting to establish the effectiveness and safety of HF therapy in clinical practice and to ascertain whether the results of a clinical trial can be extended to all individuals with HF (11, 12). This narrative review summarized published RWE studies on vericiguat, highlighting the available evidence. The robustness of the studies differed, thus precluding direct comparisons with the VICTORIA results. In fact, information from smaller studies should be interpreted carefully. Furthermore, variations in population characteristics, follow-up periods, and study endpoints hamper any direct comparison with the outcomes of the VICTORIA trial. Nevertheless, we strongly believe that the information presented may be valuable, as it consolidates all available RWE regarding vericiguat.

Our data showed that although patients were mostly older than in the VICTORIA trial, there was marked heterogeneity in the clinical profile of those taking vericiguat in clinical practice, suggesting broader applicability of the drug in diverse profiles of patients with HFrEF. Notably, unlike the inclusion criteria of VICTORIA, which required recent worsening of HF, studies from Japan and China included patients with stable chronic HF. This is important, since in the VICTORIA trial, recently decompensated HF was an inclusion criterion. Nevertheless, the novel VICTOR trial is currently analyzing the impact of vericiguat in patients with HFrEF without recently decompensated HF on a background of standard therapy (10, 41).

A high percentage of patients with HFrEF are treated with quadruple therapy in clinical practice. However, rehospitalization and decompensated HF remain unacceptably high in clinical practice, indicating that new approaches, such as vericiguat, are required (42). Although most patients in the VICTORIA trial were taking RAAS inhibitors, beta blockers, and MRA, less than 3% were taking SGLT2i. A recent review notes that traditional GDMT is often unsuitable for older patients owing to tolerability issues and comorbidities. Vericiguat has shown beneficial effects in patients with worsening HF, potentially offering advantages for elderly and frail individuals (43). RWE studies have shown that in recent years, there has been a marked increase in the use of GDMT (10, 19–30, 32, 33, 35–38, 40), consistent with the latest recommendations that encourage early use of HF drugs with proven cardiovascular benefit, even starting or up-titrating during hospitalization (6, 7, 44). Of note, in VERISEC (21) and in the study of Hashimoto et al. (29), vericiguat was started in 15–35% of patients during hospitalization, with no safety concerns. As a result, vericiguat is being added to GDMT in clinical practice, reducing HF burden thanks to the complementary mechanisms of action of vericiguat and GDMT (5). Moreover, the concomitant use of vericiguat may facilitate the optimization of other HF drugs (23, 25, 37). Therefore, in the comprehensive management of patients with HFrEF, early introduction of the 5 pillars of treatment, including vericiguat, is recommended to reduce HF burden.

In the VICTORIA trial, no significant differences were observed in the Kansas City Cardiomyopathy Questionnaire scores despite a significant reduction in HF hospitalizations with vericiguat, regardless of baseline health status (17). However, in the RWE studies that specifically analyzed this issue, an improvement was observed in both NYHA functional class and quality of life (19, 20, 22, 23, 33). Although the questionnaires used in RWE studies differed from those of the VICTORIA trial, as did the background HF therapy, the benefits shown in clinical practice were all consistent and persisted over time, thus supporting the benefits of vericiguat in improving functional status and quality of life.

In the VICTORIA trial, the addition of vericiguat significantly reduced the risk of events compared to standard therapy. However, rates of events remained high after nearly 1 year of follow-up (35.5% for the primary endpoint, 16.4% for cardiovascular death, and 27.4% for HF hospitalization) (10). By contrast, although real-world patients were older than those included in the VICTORIA trial, rates of hospitalizations for HF and deaths seemed lower in real-world populations (10, 19, 20, 22–24, 28, 31). While follow-up periods differed between the studies, the data indicate the importance of prescribing the complete GDMT, including vericiguat, in clinical practice.

LV reverse remodeling is a key therapeutic goal in HFrEF and typically occurs in response to neurohormonal modulation or specific interventions (i.e., RAAS inhibitors), although it might occur spontaneously when the cause of HF is eliminated (i.e., tachycardia-induced, alcohol-induced cardiomyopathy) (45). For example, significant reductions in volume- and diameter-related LV parameters have been observed, as has a significant increase in LVEF after 12 months of treatment with sacubitril-valsartan (46). The nitric oxide-soluble guanylate cyclase-cGMP pathway is impaired in patients with HFrEF, leading to cardiac alterations that promote the development and progression of HF. Vericiguat is an oral soluble guanylate cyclase stimulator that can help restore this pathway, promoting LV reverse remodeling (5). The prespecified echocardiographic substudy of the VICTORIA trial demonstrated a significant improvement in LV structure and function, which was confirmed by most RWE studies that assessed this issue (18, 19, 24, 28, 29, 32, 36, 39). Importantly, as no other disease-modifying HF drugs act on this pathway, the above-mentioned finding implies that vericiguat should be considered a main pillar in the comprehensive management of patients with HFrEF (4, 5).

The VICTORIA trial showed that the benefits of vericiguat over placebo in the primary outcome were obtained up to 8,000 pg/ml of NT-proBNP, indicating more advanced disease with more comorbidities and likely less benefit in affected patients (47, 48). In RWE studies, baseline NT-proBNP varied widely, up to 7,055 pg/ml. In around half of these studies, a significant reduction in natriuretic peptide levels was observed with vericiguat, although a trend or neutral effect was observed in the others (19, 22, 24, 29, 30, 32, 33). However, in some studies, the baseline levels of natriuretic peptides were higher than in the VICTORIA trial, suggesting a worse clinical profile in real-world patients (21). In any case, vericiguat is being used in clinical practice within the range of natriuretic peptides for which the VICTORIA trial showed a clinical benefit.

In the VICTORIA trial, around 90% of the patients achieved the 10 mg target dose of vericiguat, only 1.6% presented serious adverse events, and 9% had symptomatic hypotension (10). In the RWE studies, the frequency of adverse effects was also low and similar to that of the VICTORIA trial, with low discontinuation rates, indicating that vericiguat was very well tolerated in a real-world population. Nevertheless, although the clinical benefits of vericiguat persisted in clinical practice, the proportion of patients attaining the 10 mg dose in RWE studies was poorer than in the VICTORIA trial. Of note, since the maximum benefits of vericiguat were obtained with the 10 mg dose in the VICTORIA trial, more efforts should be made to titrate vericiguat adequately in clinical practice (10, 19, 20, 22–24, 28, 30, 37). In the recent VELOCITY study, which included patients with chronic HFrEF, 93% of patients safely tolerated initiation of vericiguat at the 5 mg/day dose (91% of patients in the worsening HF group and 96% in the nonworsening HF group) (49). These results could facilitate the initiation and titration of vericiguat in clinical practice.

The main strength of this study is that all the available evidence for the effectiveness and safety of vericiguat in clinical practice is summarized and critically discussed from a comprehensive point of view in different countries. However, this study has also many limitations. First, RWE is affected by a series of factors that may influence the results and restrict analyses to indirect comparisons. These include heterogeneous populations, variable follow-up periods, lack of control arms, and potential publication bias. In addition, as a control group was not available in most studies, the relative benefits of vericiguat over the standard approach could not be determined. Second, follow-up and the number of patients were limited in some RWE studies. Third, data were reported as presented in the original manuscripts. Follow-up time and event rates were available for the RWE studies, whereas only the VICTORIA trial provided standardized results per 100 patient-years, limiting the possibility of comparisons. Therefore, further specific investigations with longer follow-up and a higher number of patients would help resolve some of the hypotheses this study suggested.

In conclusion, preliminary RWE indicates that vericiguat is feasible in and can be tolerated by patients with HFrEF in clinical practice, in addition to GDMT. Reported observations include improvements in quality of life, NYHA functional class, and LV reverse remodeling. Although further data on long-term comparative effectiveness are needed, our results suggest that vericiguat could be included as part of comprehensive management for patients with HFrEF.
