# Supplementary material for: Effectiveness and safety of vericiguat in patients with heart failure and reduced ejection fraction: a narrative review of real-world evidence studies
Source: Front Cardiovasc Med. 2025 Nov 6;12:1644646. doi: 10.3389/fcvm.2025.1644646 (PMC12631807; doi:10.3389/fcvm.2025.1644646)
Supplement: Supplementary file 1 [file Supplementaryfile1.docx]

**Supplementary table 1. Changes in quality of life and functional class after treatment with vericiguat.**

| **Study** | **Results** | **Overall effect** |
| --- | --- | --- |
| **VICTORIA** | While vericiguat significantly reduced the risk of CV death and hospitalization due to HF regardless of baseline health status, no significant differences were observed in KCCQ scores between the vericiguat and placebo groups during the study. | 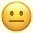 |
| **Calvo** | Greater improvements were observed in patients with worse clinical status at 3 months and at 1 year. | 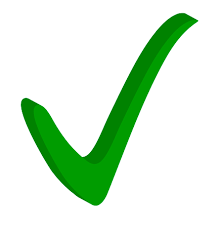 |
| **Jimenez** | At baseline, 52.4% had NYHA functional class II and 34.9% NYHA functional class III. At 6 months, these percentages were 27% and 12.7%, respectively. | 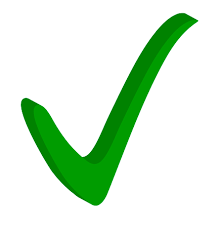 |
| **VERICIDuAT** | At baseline, 9.2%, 53.0%, 32.8%, and 5.0% had NYHA functional class I, II, III, and IV, respectively. After 162 days, these percentages were 22%, 60%, 16%, and 2%, respectively. | 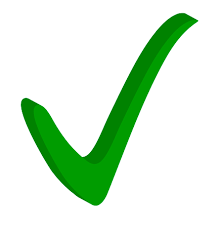 |
| **VERITA** | After a median follow-up of 303 days, NYHA functional class improved (from 67.3% and 32.7% in classes III and II, respectively, to 22.4% and 75.5% at study end; P < 0.001), as did the EQ-5D (from 0.83 ± 0.13 to 0.87 ± 0.12, P = 0.032) and VAS scores (from 60 to 79, P = 0.005). | 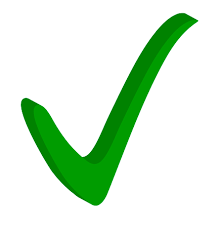 |
| **Zhao** | Significant improvements in the MLHFQ score were observed in patients treated with vericiguat in combination with GDMT for 1 month. | 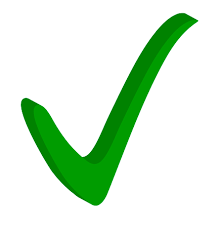 |
| **Zhan** | At 6 months, the vericiguat group exhibited a significant reduction in patients classified as C (from 31.6 to 7.5%; p<0.05) and D (from 31.6 to 3.7%; p<0.05)  in the Weber Functional Classification, compared to those patients receiving only standard therapy. | 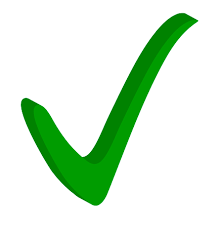 |
| **Patel** | At 6 months, the NYHA functional class improved in 41.7% of patients treated with vericiguat. This was numerically higher than the control group (26.9%). | 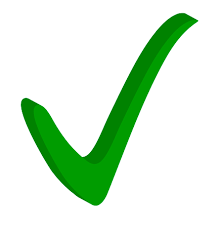 |

CV: cardiovascular; HF: heart failure; KCCQ Kansas City Cardiomyopathy Questionnaire; EQ-5D: EuroQol-5D; MLHFQ: Minnesota Living with Heart Failure Questionnaire; NYHA: New York Heart Association; VAS: visual analog scale. GDMT: guideline-directed medical therapy

Positive effect:
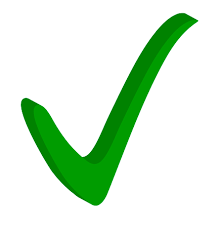
; Neutral effect:
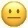
.

Table based on data from references #10, 17, 19, 20, 22, 23, 33, 36, and 40.

**Supplementary table 2. Evolution of hospitalizations for HF and mortality during follow-up after treatment with vericiguat.**

| **Study** | **Results** |
| --- | --- |
| **VICTORIA** | Over a median follow-up of 10.8 months, the primary outcome event (a composite of CV death or first hospitalization for HF) occurred in 35.5% of patients treated with vericiguat (vs 38.5% in the placebo group; HR 0.90; 95% CI 0.82-0.98; P = 0.02). CV death was recorded in 16.4% of patients in the vericiguat group (vs 17.5% in the placebo group; HR 0.93; 95% CI 0.81-1.06) and hospitalizations for HF were recorded in 27.4% of patients in the vericiguat group (vs 29.6% in the placebo group; HR 0.90; 95% CI 0.81-1.00). |
| **Calvo** | The mean number of HF decompensations decreased significantly after 3 months of treatment with vericiguat (1.55 vs 0.5; P < 0.001), with a trend after 1 year. Deaths were recorded in 5.9% of patients at 3 months and in 3.9% at 12 months. |
| **Jimenez** | At 6 months, the mortality rate was 6.7%, and 11.7% of patients were readmitted for HF. |
| **VERICIDuAT** | During the follow-up (162 days), 31% of patients required intravenous diuretics (outpatient or inpatient), and 27% visited the emergency department for decompensated HF. In relation to hospitalizations, 22% were for HF, 5% for cardiovascular causes, and 14% for noncardiovascular causes. The mortality rate was 20%. |
| **VERITA** | After a median follow-up of 303 days, the number of HF-related hospitalizations/decompensations within the previous 12 months was 2.3±1.4, decreasing with vericiguat to 0.79±1.14 (P < 0.001). At study end, 7.7% had died (50% from HF). |
| **Escobar** | After a mean duration of treatment with vericiguat of 12.4±5.3 months, 14.3% of patients had to visit the emergency department, half with hypotension and half with impaired renal function. A further 14.3% were hospitalized, half with decompensated HF. One patient (7.1%) died of sepsis. |
| **Fujii** | After 6 months, 25% of patients had experienced cardiovascular events. |
| **Chen** | This study compared the outcomes of complete standard therapy, complete standard therapy + vericiguat, incomplete standard therapy, and incomplete standard therapy + vericiguat for 180 days. The incidence of worsening HF events was lower in the 2 groups with vericiguat, and even better with incomplete therapy than with full therapy, possibly indicating that if it is difficult to start standard therapy, it would not be necessary to wait for quadruple therapy before considering adding vericiguat. |
| **Zhan** | Rehospitalization due to HF was significantly less frequent in the vericiguat group (20.0%) than in the standard treatment group (33.7%; P=0.049). |
| **Patel** | At 6 months, mortality rates were lower in the vericiguat group (10.4% vs 17.3%, *P* = 0.32), with fewer HF admissions (25.0% vs 38.5%, P = 0.14). In addition, the mean number of HF admissions per patient was significantly lower with vericiguat (0.7 vs 1.2, P = 0.03). |

CV: cardiovascular; HF: heart failure; HR: hazard ratio; CI: confidence interval.

Table based on data from references #10, 19, 20, 22-24, 28, 31, 36, 40.

**Supplementary table 3. Changes in heart structure and function, natriuretic peptides, and HF drugs and other effects observed after treatment with vericiguat.**

| **Study** | **Heart structure and function** | | | **Natriuretic peptides** | | | | | | **Concomitant HF drugs** | | | | **Other benefits** | | | | | |
| --- | --- | --- | --- | --- | --- | --- | --- | --- | --- | --- | --- | --- | --- | --- | --- | --- | --- | --- | --- |
| **VICTORIA** | After 8 months of treatment, the prespecified echocardiographic study revealed significant improvements in LV structure (reduction in LV end-systolic volume index) and function (increase in LVEF) in both the vericiguat and the placebo groups, although this tended to be more marked in patients treated with vericiguat. | | 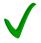 |  | | | | | |  | | | |  | | | | | |
| **Calvo** | Improvement in LVEF at 1 year of use of vericiguat (28.3 vs 34%; P = 0.025). | | 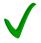 | No significant changes were observed in natriuretic peptide levels. | 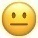 | | | | |  | | | | No changes in hemoglobin or glomerular filtration rate were observed during follow-up. | 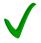 | | | | |
| **VERICIDuAT** |  | | | No significant decrease in natriuretic peptides after 162 days of follow-up. | 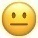 | | | | |  | | | | After 162 days of follow-up, no significant changes were observed in natriuretic peptides, hemoglobin, renal function, or systolic blood pressure. | 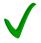 | | | | |
| **VERITA** |  | | |  | | | | | | Treatment with vericiguat enabled doses of sacubitril-valsartan to be increased significantly (P = 0.023), with a trend toward a reduction in the mean dose of furosemide. | 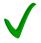 | |  | | | | | | |
| **Escobar** | No significant changes in LVEF were observed. | 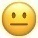 | | No significant changes in natriuretic peptide levels were observed. | | 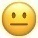 | | |  | | | | At baseline, 28.6% of patients presented NS/SVT (5–6 episodes per month). After 12 months of treatment with vericiguat, the proportion of patients with NS/SVT decreased by 50%, and a further 25% presented a substantial reduction in the number of episodes of NS/SVT. | | | 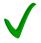 | | |  |
| **Okami** |  | | |  | | | | | | An increase in HF drug use was observed after 3 months of treatment with vericiguat. A slight decrease was observed in the use of ACEIs/ARBs. | | 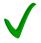 | |  | | | | | |
| **Suzuki** |  | | |  | | | | | |  | | | | Treatment with vericiguat was associated with a reduction in mean pulmonary artery pressure and pulmonary artery wedge pressure. | | | 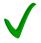 | | |
| **Fujii** | At 6 months of vericiguat use, LV reverse remodeling was observed in all patients, with significant reductions in LV end-diastolic and LV end-systolic volumes and a significant increase in LVEF (from 31.8 ± 7.4 to 37.6 ± 12.3%; P < 0.001). | 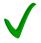 | |  | | | | |  | | | |  | | | | | |  |
| **Hashimoto** | Treatment with vericiguat was associated with a significant reduction in LV end-diastolic volume index and LV end stroke volume index and improved right ventricle-pulmonary artery coupling. | 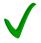 | | Vericiguat was associated with a significant reduction in natriuretic peptide levels. | | | 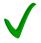 | |  | | | |  | | | | | |  |
| **Nakamura** |  | | | BNP levels tended to increase during the 6-month pretreatment period but remained unchanged after 6 months of treatment with vericiguat. | | | | 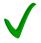 | |  | | | |  | | | | | |
| **Tian** | Vericiguat did not significantly improve LV reverse remodeling. | 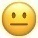 | | Vericiguat significantly reduced NT-proBNP levels. | | | 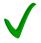 | |  | | | |  | | | | | |  |
| **Zhao** | After 1 month with vericiguat, LVEF increased significantly (from 38.1 ± 8.5% to 43.1 ± 8.5%, P < 0.01). | 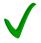 | | After 1 month with vericiguat, NT-proBNP levels decreased significantly (from 4567.8 ± 5163.9 to 1895.6 ± 2702.1 ng/L, P < 0.01). | | | 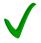 | |  | | | |  | | | | | |  |
| **Zhan** | Compared to the standard treatment group, patients treated with vericiguat showed significant improvement in LVEF and LV end-diastolic diameter at 3 and 6 months. Additionally, main pulmonary artery diameter improved as early as 1 month after treatment with vericiguat. | 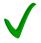 | |  | | | | |  | | | | Compared to the control group, vericiguat significantly increased peak oxygen consumption and anaerobic threshold and reduced the carbon dioxide equivalent slope. | | | | | 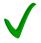 |  |
| **Kerwagen** |  | | |  | | | | | | The percentage of patients receiving quadruple guideline-directed medical therapy increased from 29% before initiation of vericiguat to 44% afterwards. | | 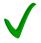 | |  | | | | | |
| **Natale** |  | | |  | | | | | |  | | | | Treatment with vericiguat was associated with a significant reduction in the renal arterial resistance index. | 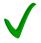 | | | | |
| **Poglajen** | Vericiguat therapy could improve right ventricular function in patients with advanced HF and left ventricular assist device support. | 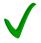 | |  | | | | |  | | | |  | | | | | |  |
| **Patel** | LVEF improved more in the vericiguat group than in the control group (+5.1% vs +2.3%, P < 0.01). | 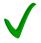 | |  | | | | |  | | | |  | | | | | |  |

ACEI: angiotensin-converting-enzyme inhibitors; ARB: angiotensin II receptor blockers; BNP: plasma B-type natriuretic peptide; HF: heart failure; LV: left ventricle; LVEF: left ventricular ejection fraction; NS/SVT: nonsustained/sustained ventricular tachycardia. NT-proBNP: N-terminal pro b-type natriuretic peptide. Positive effect:
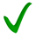
; Neutral effect:
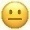
. Table based on data from references #17-25, 27-30, 33, 33, 36-40.

**Supplementary table 4. Safety of vericiguat.**

| **Study** | **Dose** | **Adverse events** |
| --- | --- | --- |
| **VICTORIA** | After approximately 12 months, 90.3% of the patients were receiving the 10-mg target dose. | At least 1 adverse event in 32.8% of patients, although only serious in 1.6%. Symptomatic hypotension was reported in 9.1% of patients, syncope in 4.0%, and systolic blood pressure declined slightly in both groups over the first 16 weeks. |
| **Calvo** | Vericiguat 10 mg was attained in 50.0% of patients at 3 months and in 53.8% at 12 months. | Vericiguat was withdrawn in 11.8% of patients at 3 months and in 5.9% at 12 months. Symptomatic hypotension was recorded in 15% of patients. |
| **Jimenez** | At 6 months, vericiguat 10 mg was reached in 75% of patients and 5 mg in 18.7%. | Adverse events occurred in 1.7% of patients, no syncope was reported, and 6.8% discontinued treatment. |
| **VERICIDuAT** | At study end, 71% of patients reached the 10-mg dose and 21% the 5-mg dose. | Vericiguat was discontinued by 11%. Discontinuation was due to hypotension in 39%, pruritus in 23%, and digestive problems in 23%. |
| **VERITA** | Target dose of 10 mg reached by 78.8% of patients. | After a median follow-up of 303 days, 13.5% had symptomatic hypotension, and 11.5% discontinued treatment (9.6% because of hypotension). |
| **Escobar** | At 12 months, 50.0% of patients achieved the target dose of 10 mg daily. | Vericiguat was discontinued because of hypotension in 7.1%. |
| **Fujii** | At 6 months, 49% reached the 10-mg dose of vericiguat. |  |
| **Nakamura** |  | After a median follow-up of 236 days, 10.7% of patients discontinued vericiguat because of symptomatic hypotension. |
| **Tian** |  | After 6 months of follow-up, the incidence of mild to moderate gastrointestinal symptoms was higher in the vericiguat group than in the control group (23.62 % vs. 2.74 %, P < 0.001). Severe hypotension was reported by 12.6% of patients in the vericiguat group and 8.2% in the control group (P = 0.341). No differences were observed in syncope rates (0.79% vs 0% P = 1.0). |
| **Rao** |  | A total of 617 vericiguat-related adverse event reports were identified, with the most frequently reported preferred terms hypotension (n = 86), dizziness (n = 52), malaise (n = 25), decreased blood pressure (n = 23), and anemia (n = 21). |
| **Zhan** |  | At 6 months, 6.2% of patients treated with vericiguat presented hypotension. |
| **Kerwagen** | Although around 70% of patients were up-titrated beyond 2.5 mg, only 36% reached the 10-mg dose (median time to up-titration from 2.5 mg to 5 mg 17 days, and from 2.5 to 10 mg 37 days), with high adherence (87%). |  |
| **Patel** |  | Hypotension was slightly less frequent in the vericiguat group (6.3% vs 7.7%, *P* = 0.78), with comparable rates of renal dysfunction, hyperkalemia, dizziness, gastrointestinal issues, and fatigue. |

Table based on data from references #10, 19, 20, 22-24, 28, 30, 32, 34, 36, 37, 40.
